# Preliminary Results about Lamb Meat Tenderness Based on the Study of Novel Isoforms and Alternative Splicing Regulation Pathways Using Iso-seq, RNA-seq and CTCF ChIP-seq Data

**DOI:** 10.3390/foods11081068

**Published:** 2022-04-07

**Authors:** Zehu Yuan, Ling Ge, Weibo Zhang, Xiaoyang Lv, Shanhe Wang, Xiukai Cao, Wei Sun

**Affiliations:** 1 Joint International Research Laboratory of Agriculture and Agri-Product Safety of Ministry of Education, Yangzhou University, Yangzhou 225000, China; yuanzehu@yzu.edu.cn (Z.Y.); dx120170085@yzu.edu.cn (X.L.); cxkai0909@163.com (X.C.); 2 College of Animal Science and Technology, Yangzhou University, Yangzhou 225000, China; mx120200799@stu.yzu.edu.cn (L.G.); mz120191042@stu.yzu.edu.cn (W.Z.); 007121@yzu.edu.cn (S.W.)

**Keywords:** sheep, tenderness, novel isoforms, alternative splicing, CTCF, Iso-seq, ChIP-seq

## Abstract

Tenderness is an important indicator of meat quality. Novel isoforms associated with meat tenderness and the role of the CCCTC-binding factor (CTCF) in regulating alternative splicing to produce isoforms in sheep are largely unknown. The current project studied six sheep from two crossbred populations (Dorper × Hu × Hu, DHH and Dorper × Dorper × Hu, DDH) with divergent meat tenderness. Pooled Iso-seq data were used to annotate the sheep genomes. Then, the updated genome annotation and six RNA-seq data were combined to identify differentially expressed isoforms (DEIs) in muscles between DHH and DDH. These data were also combined with peaks detected from CTCF ChIP-seq data to investigate the regulatory role of CTCF for the alternative splicing. As a result, a total of 624 DEIs were identified between DDH and DHH. For example, isoform 7.524.18 transcribed from *CAPN3* may be associated with meat tenderness. In addition, a total of 86 genes were overlapped between genes with transcribed DEIs and genes in differential peaks identified by CTCF ChIP-seq. Among these overlapped genes, *ANKRD23* produces different isoforms which may be regulated by CTCF via methylation. As preliminary research, our results identified novel isoforms associated with meat tenderness and revealed the possible regulating mechanisms of alternative splicing to produce isoforms.

## 1. Introduction

Meat tenderness is determined by several intrinsic factors (e.g., proteolytic activity, amount of glycogen, fiber type and connective tissue) and extrinsic factors (e.g., animal breed and nutritional conditions) [1]. Among these factors, the calpain system plays an essential role in meat tenderness [2]. In beef, the most important factor that determines tenderness is the proteolytic activity of the calpain system. Calpain3 (CAPN3), a member of the calpain system, is mainly expressed in skeletal muscle, especially in type II fibers [3,4,5], and it may play an important role in the calpain system to regulate meat tenderness [6]. A previous study suggested that the expression level of CAPN3 protein and mRNA was significantly associated with tenderness [7,8]. Another member of the calpain system is calpastatin (*CAST*). Accumulated evidence suggests that different isoforms transcribed from a gene due to alternative splicing may play different biological roles. For example, distinct isoforms due to alternative splicing [9] in *CAST* have different biological functions in beef meat tenderness.

PacBio long-reads isoform sequencing (Iso-seq), a third-generation sequencing technology, can directly obtain full-length isoforms [10]. By combing with traditional RNA-sequencing (RNA-seq), Iso-seq can contribute to identify novel isoform associated with complex traits [11,12,13]. Although differentially expressed gene expression studies have been implemented for meat quality traits in sheep [14,15], studies focusing on the investigation of novel isoforms in sheep muscle are still scarce. Alternative splicing is the main mechanism to diversify isoforms and is regulated by numerous interacting components [16]. For example, the CCCTC-binding factor (CTCF) has been identified as a modulator of the alternative splicing process [17]. However, the role of CTCF in regulating alternative splicing to produce isoforms in sheep is largely unknown. In this context, using Iso-seq, RNA-seq and CTCF chromatin immunoprecipitation sequencing (ChIP-seq) data in sheep muscle can help to identify key isoforms associated with meat tenderness and to reveal the biological mechanisms of alternative splicing regulation to produce isoforms.

Tenderness is an important indicator of meat quality with the greatest consumer appreciation [18]. Crossbreeding is a common method for increasing sheep production. When using crossbreeding methods to increase sheep meat production, some meat quality traits, e.g., tenderness, show a significant difference across different cross-breed populations. Unveiling the genetic factors associated with tenderness is essential for guiding cross-breeding efficiently and profitably. Therefore, revealing the genetic processes that regulate meat quality between different cross-breeds is of great interest to the sheep industry.

The goals of the current study were to identify key isoforms associated with meat tenderness and to reveal the biological mechanisms of alternative splicing regulations to produce isoforms. To achieve these goals, differentially expressed isoforms (DEIs) were identified between two cross-bred populations with divergent meat tenderness by integrating Iso-seq and RNA-seq data. Then, we combined peaks detected from CTCF chromatin immunoprecipitation sequencing (ChIP-seq) data with DEIs to investigate the possible mechanism with which CTCF regulates the alternative splicing process. Our results have the potential to reveal novel isoforms associated with meat tenderness and uncover the possible regulating mechanism of alternative splicing to produce isoforms.

## 2. Materials and Methods

**Figure 1:**
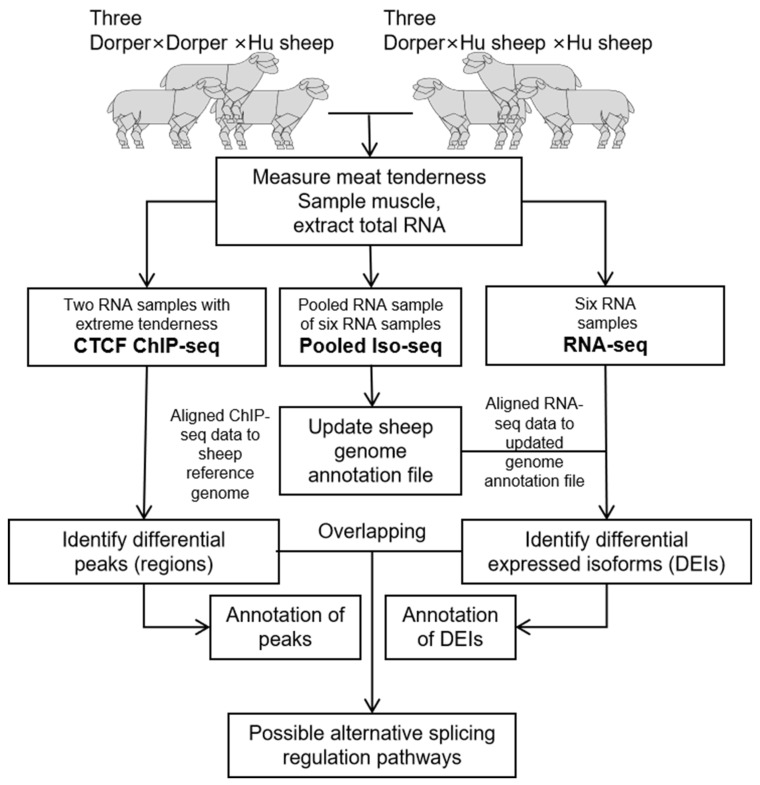
The overall experimental design. DEIs, differentially expressed isoforms.

The animal experiment of this study was approved by the Experimental Animal Ethical Committee of Yangzhou University (File NO. 202103294). The overall experimental design of the current study is shown in Figure 1.

### 2.1. Animal Samples and Meat Traits

In the current study, a total of six unrelated 6-month-old male sheep, including three Dorper × Hu × Hu (DHH) and three Dorper × Dorper × Hu (DDH), were selected from a large population for sampling and measuring meat tenderness. Six male sheep were fed in the same environment from 2 months old to 6 months old. Six sheep were selected with similar live weights (DHH = 40.80 ± 4.57 kg, DDH = 41.50 ± 0.95 kg, Table 1) to rule out the effect of live weight on meat tenderness. Live weight was measured before slaughter (24 h in advance). After slaughter, *longissimus dorsi* from between the 12th and 13th ribs from six sheep were sampled within 30 min. Each muscle sample was packed into a 1.5 mL cryotube with triplicate. All the muscle samples were frozen in liquid and stored in a −80 °C refrigerator. After slaughter, carcass weight was measured by an electronic scale. Then, 45 min after slaughter, *longissimus dorsi* were cut after the 12th rib and the samples were cooked for 35 min at 71 °C [19]. Then, six 1 cm^2^ cylinders were cut and all samples were tested using a meat tenderness analyzer (CL3, NanJing, CN). The difference of meat tenderness between DDH and DHH was tested by the *t*-test function in the basic environment of R 4.1.1 [20]. A *p* value smaller than 0.05 denotes significance.

### 2.2. RNA-Extraction, RNA-seq and Iso-seq

A Trizol reagent Kit (TaKaRa, Kusatsu, Shiga, Japan) was used to extract the total RNA from six muscle tissues. Then, Nanodrop 2000 (Thermo Fisher Scientific, Waltham, MA, USA) and a 2100 Bioanalyzer (Agilent Technologies, Waldronn, Germany) were used to evaluate the RNA Integrity Number (RIN >7 passed quality control) and 28S/18S ratio (ratio > 1.0 passed quality control). After quality control of all six RNA samples, they were used for library construction. Following library quality control, six libraries were sequenced on the MGISEQ-2000 (BIG) platform [13].

Pooled RNA samples of six muscle tissues were used for Iso-seq. Briefly, cDNA was transcribed from a pooled RNA sample using the SMARTerTM PCR cDNA synthesis kit (Takara Biotechnology, Dalian, China). Following PCR amplification, PCR product purification, size selection (>1 kb), SMRTbell library construction, and library quality control, the library was sequenced on the PacBio sequencing platform. The raw Iso-seq data were processed using the SMRT Link v8.0 pipeline. Briefly, the BAM file was processed to obtain the circular consensus sequence (CCS). Full-length CCS reads with 5′ and 3′ cDNA primers and polyA were defined as full-length non-concatemer (FLNC) reads. FLNC reads were corrected by six clean RNA-seq data using LoRDEC v0.9 software [21]. The quality control of six RNA-seq data was implemented by SOAPnuke v2.1.0 [22].

### 2.3. Update Sheep Reference Annotation File

The pooled Iso-seq data were used to update the sheep reference annotation file. After correction, FLNC reads were mapped to the reference genome (http://ftp.ensembl.org/pub/release-102/fasta/ovis_aries_rambouillet/dna/, accessed on 29 March 2022) using GMAP [23]. Isoforms that met at least one of the following criteria were kept: first, an isoform was identified by at least two FLNC reads; second, an isoform was identified by only one FLNC read with a percentage identity (PID) of greater than 99%; third, all splicing sites in an isoform were identified by RNA-seq data; fourth, all alternative splicing events in a identified isoform were also annotated by the reference genome annotation file. Isoforms that overlapped less than 20% of their length on the same strand were identified as distinct isoforms. A novel gene was defined as a gene locus overlapping less than 20% of their length with known genes. An isoform with a new emerged intron (exon) or a final splice site of 3′ changed ends was defined as a novel isoform. In addition, the alternative splicing events were detected by the AStalavista v3.2 software [24].

### 2.4. Differentially Expressed Isoform Detection and Functional Analyses

Bowtie2 was used to align short reads to novel annotation files [25]. The DESeq2 R Bioconductor package was used to identify DEIs between two groups [26]. An isoform with a fold change (FC) greater than two and a false discovery rate (FDR) smaller than 0.05 was defined as a significant DEI. All identified DEIs were blasted to GO database using Diamond [27] and blasted to the KEGG database using KOBAS [28] to extract their potential biological functions. The GOplot 1.0.2 R package was used to visualize GO enrichment results [29]. GO terms and KEGG pathways with an FDR smaller than 0.05 were regarded as having significant enrichment. 

### 2.5. Validation of Differentially Expressed Isoforms

The isoform abundance of five DEIs were quantified for validating the result of identified DEIs by quantitative real-time PCR (qRT-PCR). Five isoform-specific paired premiers were designed by SnapGene^®^ v2.3.2 software (from Insightful Science; available at snapgene.com) and were synthesized by Tsingke Biotechnology Co., Ltd. (Nanjing, China, Table S1). cDNA was synthesized using a FastKing gDNA Dispelling RT Super Mix (TIANGEN, Beijing, China). The qRT-PCR was performed on a CFX96 Connect™ Real-Time System (BIO-RAD, Hercules, CA, USA) using a 20 μL reaction volume, including 1 μL of cDNA in 10 μL of 2× TSINGKE Master qPCR Mix (SYBR Green I) (Tsingke, Nanjing, China), 0.8 μL (10 μm/μL) each of the forward and reverse primers, and 7.4 μL of distilled water. The abundance of the *GAPDH* was used as the control. Each biological sample was implemented in triplicate, and the 2−ΔΔCt method was used for calculating the relative expression level of isoforms.

### 2.6. CTCF ChIP-seq

We aimed to explore the possible regulating mechanism of CTCF in alternative splicing in sheep muscle. Two muscle samples with extreme meat tenderness were selected to implement CTCF ChIP-seq. CTCF ChIP-seq was implemented by a commercial sequencing provider (igenebook Technology Co., Ltd., Wuhan, China). Briefly, chromatin was crosslinked by formaldehyde, following nuclear processing, chromatin digestion, DNA-protein compound capture, decrosslinking of DNA-protein compounds, and the purification of DNA. Finally, the input and ChIP DNA samples were sequenced on the Illumina Hiseq X ten platform.

### 2.7. Bioinformatics Analysis of CTCF ChIP-seq

The quality of CTCF ChIP-seq data was evaluated by fastqc v0.11.5 [30]. The quality control of the raw CTCF ChIP-seq data was implemented by Trimmomatic v0.36 [31]. After quality control, clean reads were aligned to the sheep reference genome by BWA v0.7.1 [32]. The read distribution in different genomic regions was investigated using the ChIPseeker R Bioconductor package [33]. An Upset plot was plotted by the UpSetR R package [34]. Potential peaks were called by MACS v2.1.1 [35]. Differential peaks were detected by DiffBind v1.16.3 (https://bioconductor.org/packages/release/bioc/html/DiffBind.html, accessed on 29 March 2022). Peaks with an FDR < 0.05 and a Fold value >0 were defined as significant differential peaks. Motifs in significant differential peaks were predicted by HOMER v3 [36]. 

### 2.8. Overlapping between DEIs and Differential Peaks Called from ChIP-seq Data

The overlapping between genes with transcribed DEIs and genes located in differential peaks were investigated to explore the regulating role of CTCF in the alternative splicing process. The significance of overlapping was tested by the GeneOverlap R Bioconductor package (https://bioconductor.org/packages/release/bioc/html/GeneOverlap.html, accessed on 29 March 2022).

## 3. Results

### 3.1. Meat Traits

In the current study, a total of three meat traits were measured after slaughter. The mean value and standard deviation of three meat traits are documented in Table 1. Among these traits, shear force in DDH was significantly (*p* = 0.0406) higher than that in DHH. Other meat traits did not show a significant difference between DDH and DHH.

### 3.2. Update of Reference Genome Annotation File

**Figure 2:**
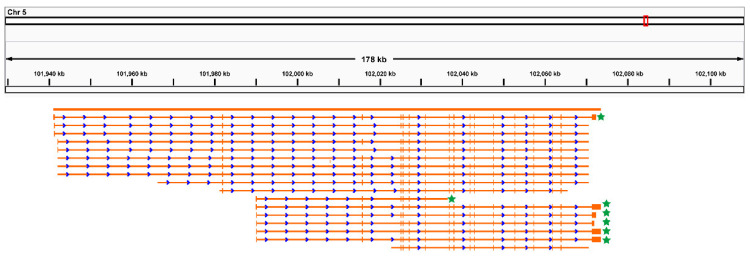
Identified isoforms in *CAST*. The numbers at the top of the figure represent the genome coordinates. Each yellow horizontal line represents an isoform. Each yellow vertical dash represents an exon. Stars on the right denote the novel isoforms identified in the current study. Blue arrows denote the direction of transcription. Stars in right denote novel isoforms.

To update the sheep genome annotation (Generic Feature Format, GFF), which is essential for accurately quantifying the abundance of isoforms, six RNA samples were pooled to implement Iso-seq. In total, 442,966 polymerase reads were produced by pooled Iso-seq (Table S2). After pre-processing, a total of 247,201 FLNC reads were kept for further analysis (Table S2). Novel genes and isoforms were identified according to the genomic position of each FLNC. In total, 18,959 gene loci (9184 known and 9775 novel) were identified (Figure S1a). Among these detected genes, 5104 (26.92%) loci had two or more transcripts (Figure S1b). In sheep muscle, 20,205 novel isoforms (57.51%) were transcribed from annotated genes (Figure S1c), followed by those transcribed from novel genes. Identified potential novel gene loci and novel isoforms as well as sheep reference annotation files (GFF file) were merged to obtain an updated genome annotation file (Table S3). Alternative splicing is the main mechanism to diversify isoforms. In this study, a total of 38,070 alternative splicing events were detected by pooled Iso-seq data (Figure S2). For example, seven novel isoforms in six pooled samples were identified in *CAST* due to alternative splicing (Figure 2).

### 3.3. Differentially Expressed Isoforms and Functional Analysis

**Figure 3:**
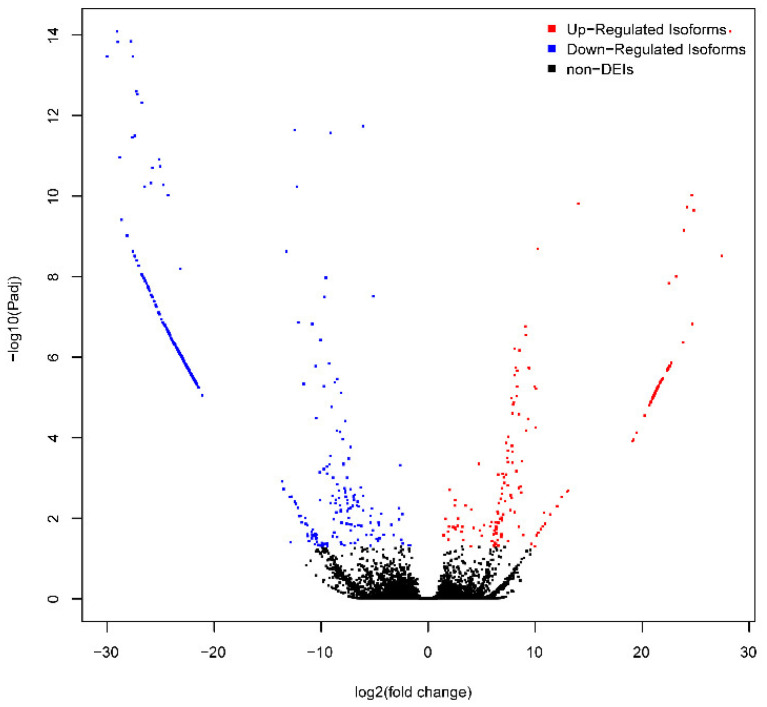
Volcano plot of differentially expressed isoforms (DEIs). *X*-axis denotes log2 (fold change). *Y*-axis denotes −log10 (adjusted *p* value). Red dots denote the up-regulated isoforms. Blue dots denote down-regulated isoforms.

Six RNA-seq data were aligned to the updated genome annotation file (Table S3) to identify the DEIs between DDH and DHH. As a result, a total of 624 DEIs were identified (Figure 3, Table S4). These 624 DEIs were transcribed from 492 genes, suggesting that some genes could produce more than one DEI. The most significant DEIs were X.351.3 *(FHL1*, FDR = 8.21 × 10^−15^) and 11.673.91 (*MYH2*, FDR = 8.21 × 10^−15^). 

**Figure 4:**
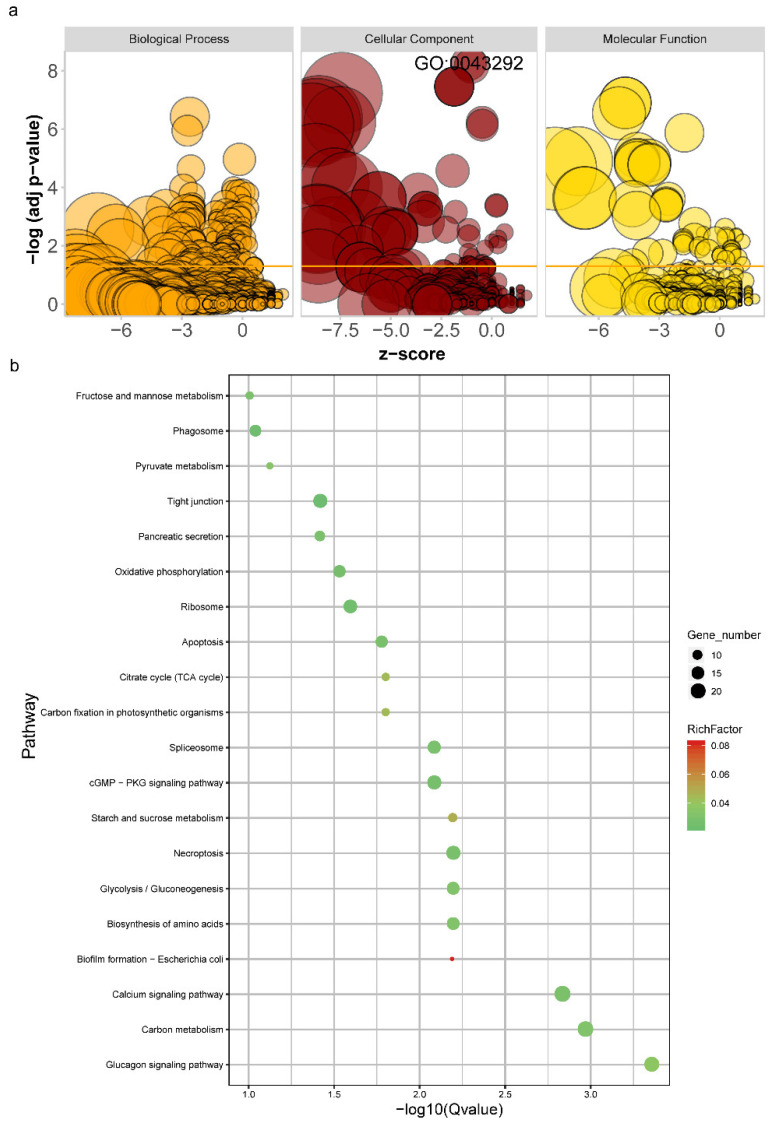
Functional annotation of differentially expressed isoforms (DEIs); (**a**) gene ontology (GO) enrichment analysis; *X*-axis denotes z-score; *Y*-axis denotes -log10(adjusted *p* value); bubble size denotes the enriched gene numbers; (**b**) Kyoto Encyclopedia of Genes and Genomes (KEGG) enrichment analysis; *X*-axis denotes −log10 (adjusted *p* value); *Y*-axis denotes enriched pathway; bubble size denotes the enriched gene numbers; bubble color denotes the adjusted *p* value.

All detected DEIs were significantly (FDR < 0.05) enriched in 280 GO terms (Figure 4a, Table S5). The most significant GO term was contractile fiber (GO:0043292, FDR = 5.38 × 10^−9^, Figure 4a, Table S5). All detected DEIs were significantly (FDR < 0.05) enriched in 17 KEGG pathways (Figure 4b, Table S6). The most significant pathway was the glucagon signaling pathway (ko049222).

### 3.4. Validation for Target Isoforms

**Figure 5:**
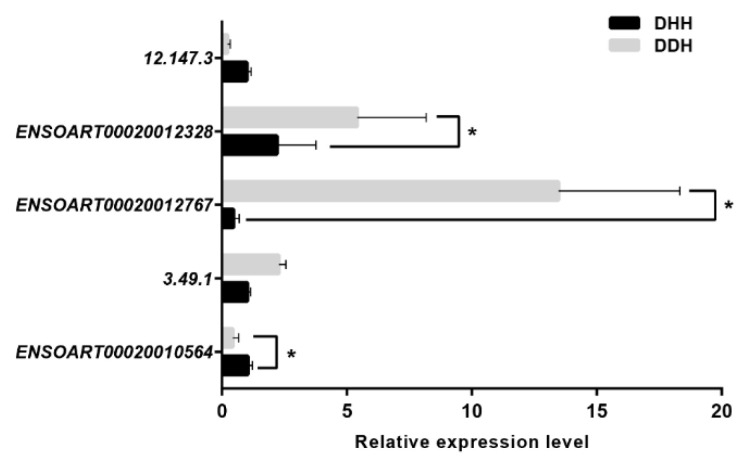
Validation of the differentially expressed isoforms by qRT-PCR; *Y*-axis denotes isoforms; *X*-axis denotes isoform relative expression level; DDH denotes Dorper × Dorper × Hu; DHH denotes Dorper × Hu × Hu. Results were presented as the mean ± SEM, * *p* < 0.05, significant difference.

To validate the result of the identified DEIs, the qRT-PCR of five DEIs was implemented. Among these five isoforms, three DEIs identified from the transcriptome analysis were significantly expressed (Figure 5). The expression trends of these five isoforms, determined by qRT-PCR, were consistent with the transcriptome data.

### 3.5. CTCF ChIP-seq

**Figure 6:**
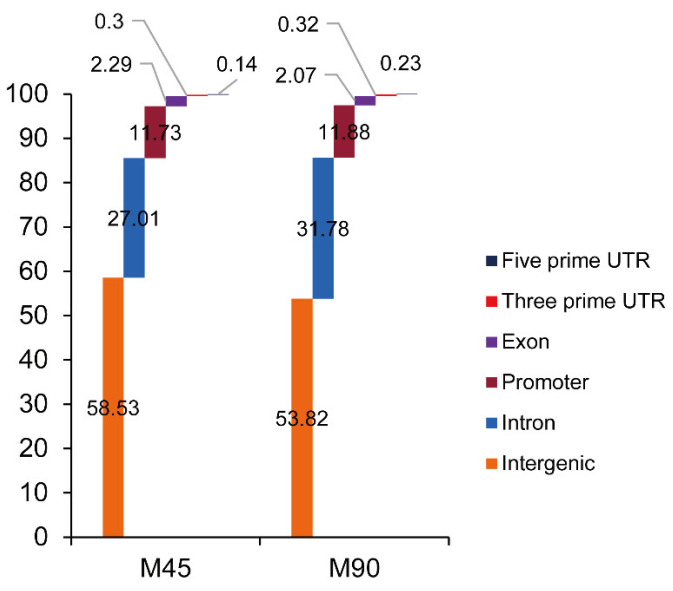
Read distribution of CTCF chromatin immunoprecipitation sequencing (ChIP-seq) among sheep genomes. According to the genome coordinates of reads, reads can be divided into six categories, including in intergenic region, intron region, promoter region, exon region, 3′ untranslated region (UTR) and 5′ untranslated region.

Two muscle samples with divergent meat tenderness were selected for CTCF ChIP-seq. After quality control, more than 40 M clean reads in each sample were obtained (Table S7). All clean reads were mapped to the sheep reference genome with a mapping rate greater than 96.46% in each sample (Table S7). Above half of the mapped reads were located in the intergenic region (Figure 6), followed by an intron, promoter, exon, 3′ untranslated regions (UTR) and 5′ UTR.

**Figure 7:**
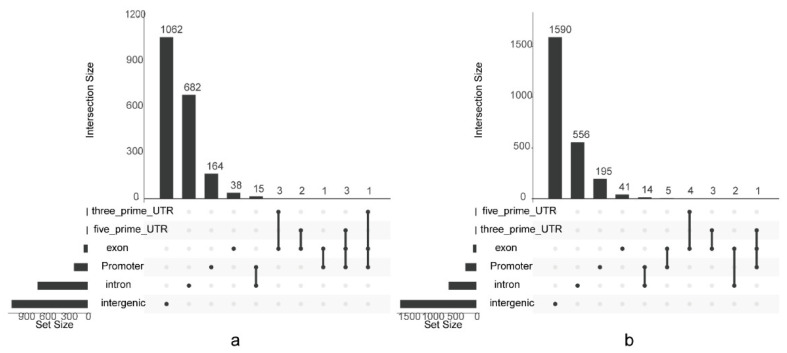
Distribution of differential peaks among the sheep genomes. (**a**) Up-regulated peaks; (**b**) down-regulated peaks. According to the genome coordinates of detected peaks, peaks can be divided into six categories, including in intergenic region, intron region, promoter region, exon region, 3′ untranslated region (UTR) and 5′ untranslated region. Dots in the bottom denote the peak categories. Black lines connecting the dots show peak numbers shared among several categories. The number above each bar shows the number of peaks for each category or shared category.

A total of 4388 differential peaks were detected between two samples with divergent meat tenderness (Table S8). The greatest number of differential peaks were distributed in the intergenic genome region, followed by the intron region, promoter region, exon region, 5′ UTR and 3′ UTR (Figure 7). The motifs in the differential peaks were predicted. A total of 66 motifs were predicted in differential peaks (Table S9).

### 3.6. Overlap between DEIs and Differential Peaks

**Figure 8:**
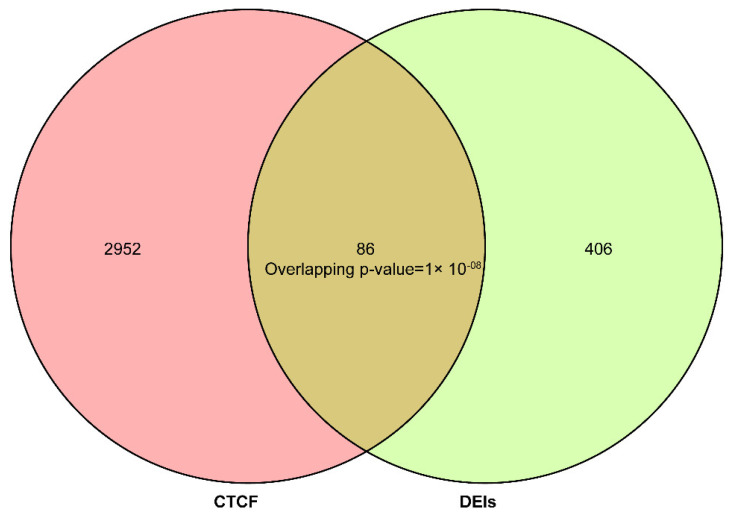
Veen plot of genes in differential peaks (**left**) and genes with transcribed differentially expressed isoforms (DEIs, **right**).

To investigate the potential role of CTCF in regulating alternative splicing in sheep muscle, the overlapping analysis between genes with transcribed DEIs and genes located in differential peaks was implemented. A total of 86 overlapped genes were found (Figure 8). The overlapping *p*-value was smaller than 0.05, indicating that the number of overlapped genes was too great to have been by chance.

## 4. Discussion

### 4.1. Update Sheep Reference Genome Annotation File

It has been reported that one possible method to quantify transcripts is mapping the reads to the annotated transcriptome [37]. In this study, pooled Iso-seq data were used to update the reference genome annotation file. As a result, 9775 possible novel genes and 30,513 novel isoforms were identified (Figure S1) in sheep muscle and were added to the sheep reference genome annotation file (Table S3). In a pig study, a total of 10,465 novel genes were identified by integrating Iso-seq and RNA-seq data [38]. Similarly, in sheep tail fat, a total of 9001 novel genes and 36,667 novel isoforms were detected using pooled Iso-seq data [13]. Our results are in line with these published works, which suggests that Iso-seq is a useful method to improve genome annotation in sheep. 

Alternative splicing is the main mechanism to diversify isoforms. Here, we highlight an example of an alternative splicing event that may regulate meat tenderness. *CAST* is linked with meat tenderness across many farm animals [39]. In beef, it has been reported that alternative splicing [9] in *CAST* could regulate meat tenderness. In sheep, a previous study suggested that SNPs in *CAST* may regulate exon excision events in *CAST* (Oar.3.1, Chr5:93439378-93444596), which was identified by RNA-seq [40]. In the current study, this exon excision event was further validated by Figure 2. Taken together, all of these pieces of evidence indicate that alternative splicing in *CAST* regulated by SNPs may play an important role in the meat tenderness of sheep.

### 4.2. Novel Isoforms Linked with Meat Tenderness

The calpain system plays an essential role in meat tenderness [2]. Calpain3 (CAPN3), a member of the calpain system, is mainly expressed in skeletal muscle and may play an important role in the calpain system to regulate meat tenderness [6]. In the current study, a novel transcript, 7.524.18, transcribed from *CAPN3,* was identified as a DEI (Table S4), suggesting its potential role in meat tenderness.

Muscle fiber type also plays an important role in meat tenderness [41,42]. Muscle fiber type is related to the abundance of myosin type [43]. The ratio of slow and fast heavy chain myosin is linked to the ratio of type I and type II fibers [44]. In the current study, 12 DEIs transcribed from four myosin heavy chain genes were identified (11.673.36, 11.673.38, 11.673.91 and 11.673.53 transcribed from *MYH2*; ENSOART00020028698 transcribed from *MYH4*; 7.469.116, 7.469.121 and 7.469.129 transcribed from *MYH7*; 14.723.9, 14.723.27, 14.723.58 and 14.723.65 transcribed from *MYBPC2*; Table S4). Previous studies have reported that *MYH2*, *MYH4*, *MYH7* and *MYBPC2* play an important role in degerming muscle fiber types [45,46]. Overall, the myosin heavy chain-related isoforms were differentially expressed between DDH and DHH, suggesting that these isoforms may regulate meat tenderness directly or indirectly.

In addition, the amount of glycogen could affect meat tenderness [47]. In the current study, seven DEIs transcribed by two glycogen-related genes were detected (14.271.7 transcribed from *GYS1*; 21.138.57, 21.138.27, 21.138.13, 21.138.58, 21.138.9 and ENSOART00020017812 transcribed from *PYGM*). A previous study suggested that *PYGM* is related to shear force in cattle [48]. These DEIs might regulate tenderness by controlling glycogen content in muscles.

GO and KEGG enrichment analyses were implemented to extract the potential function of DEIs. In pigs, differentially expressed genes between fast and slow muscle were relevant to myofibril and contractile fiber GO terms. In the current study, DEIs were most significantly enriched in contractile fiber (GO:0043292) and myofibril (GO:0030016) GO terms. Our results provide further evidence for a relationship between muscle fiber type transformation, meat tenderness and DEIs. For the KEGG pathway enrichment analysis, a previous study suggested that a gene in the calcium signaling pathway (ko04020) is related to stiffness and affects the speed of fiber degradation during the meat aging process [49]. In the current study, 23 DEIs were significantly enriched in calcium signaling pathways, indicating their potential role in the meat aging process. 

### 4.3. CTCF Might Regulate Alternative Splicing in Sheep Muscle

In the current study, CTCF ChIP-seq was implanted to investigate its potential role in regulating alternative splicing in sheep muscle with divergent meat tenderness. The main function of alternative splicing processes are to diversify isoforms [50]. In recent years, CTCF has been identified as a modulator of the alternative splicing process [17]. In this study, 86 overlapped genes were found between genes transcribing DEIs and genes in CTCF peaks, and the number of overlapped genes was too great to be accidental (Figure 7). These results suggest that CTCF may regulate alternative splicing in sheep muscle. 

The CTCF-regulated alternative splicing mechanism can be divided into co-transcriptional, genomic and epigenetic mechanisms [17]. Here, our study example suggests that CTCF may regulate alternative splicing through the CTCF-mediated DNA methylation process in sheep muscle. In the current study, two DEIs, 3.1299.2 and 26.152.10, were transcribed from *ANKRD23* (Table S4). In a cattle study, the ANKRD23-202 mRNA isoform, which is a splice form of the ANKRD, was defined as a DEI between the tough group and the tender group [49]. The result of a rabbit study suggested that A*NKRD23* methylated in promoter and gene body regions is associated with exon skipping alternative splicing in the skeletal muscle [51]. Previous results suggest that CTCF could regulate alternative splicing by controlling DNA methylation [52,53,54]. In the current study, a CTCF peak was observed in *ANKRD23* (Table S8). Taken together, CTCF may regulate *ANKRD23* to produce different isoforms mediated by methylation.

As the first analysis of lamb meat tenderness based on the study of novel isoforms and alternative splicing regulation pathways, our study has its limitations. In our study, the sample size was relatively small, which may reduce the power to detect DEIs. In addition, tenderness is a parameter linked to DNA, exposition, individual characteristics, and a multitude of other factors that genetics cannot fully explain. Thus, in the future, we should increase sample size and consider more factors to investigate lamb meat tenderness. Nevertheless, our results provide the first insights about lamb meat tenderness based on the study of novel isoforms and alternative splicing regulation.

## 5. Conclusions

In this study, a total of 624 DEIs were identified between DDH and DHH. For example, isoform 7.524.18 transcribed from *CAPN3* may be associated with meat tenderness. In addition, a total of 86 overlapped genes were found between genes with transcribed DEIs and genes in differential peaks identified by CTCF ChIP-seq. Among overlapped genes, *ANKRD23* produces different isoforms that may be regulated by CTCF mediated by methylation. As preliminary research, our results identified novel isoforms associated with meat tenderness and revealed the possible regulating mechanism of alternative splicing to produce isoforms using bioinformatic analysis. In the future, more samples should be collected and more molecular experiments, e.g., TA clone, ChIP-qPCR, etc., should be implemented to improve these preliminary results.

## Figures and Tables

**Table 1:** Meat traits of Dorper × Hu × Hu (DHH) and Dorper × Dorper × Hu (DDH) sheep.

Meat Trait (Unit)	Crossing Type		Statistic (t)	*p* Value
	DDH ^1^ (n = 3)	DHH ^2^ (n = 3)		
Live weight (kg)	40.80 ± 4.57	41.50 ± 0.95	−0.2720	0.8094
Carcass weight (kg) ^3^	22.00 ± 2.27	23.70 ± 0.64	−1.2717	0.3161
Shear force (N) ^3^	83.20 ± 13.60	53.30 ± 8.39	3.2558	0.0406

^1^ DDH denotes Dorper × Dorper × Hu. ^2^ DHH denotes Dorper × Hu × Hu. ^3^ These traits were measured on hot carcass.

## Data Availability

All of the raw sequencing data will be available through NCBI SRA: PRJNA745517.

## Supplementary Materials

The following supporting information can be downloaded at: https://www.mdpi.com/article/10.3390/foods11081068/s1: Figure S1: Identified novel genes and isoforms: (a) Venn plot of identified genes and annotated genes; (b) frequency of the number of transcripts transcribed by a gene; (c) classification of identified isoforms; Figure S2: Classification of alternative splicing events; Table S1: Designed isoform-specific primers; Table S2: Data information of pooled isoform sequencing (Iso-seq) in sheep muscle; Table S3: Updated sheep genome annotation file; Table S4: Differentially expressed isoforms; Table S5: Gene ontology (GO) enrichment analysis of differentially expressed isoforms; Table S6: Kyoto Encyclopedia of Genes and Genomes (KEGG) enrichment analysis of differentially expressed isoforms; Table S7: Summarized information of chromatin immunoprecipitation sequencing (ChIP-seq); Table S8: Differential peaks between two muscle samples with divergent tenderness; Table S9: Predicated motifs in differential peaks.
