# Supplementary material for: Preliminary Results about Lamb Meat Tenderness Based on the Study of Novel Isoforms and Alternative Splicing Regulation Pathways Using Iso-seq, RNA-seq and CTCF ChIP-seq Data
Source: Foods. 2022 Apr 7;11(8):1068. doi: 10.3390/foods11081068 (PMC9025809; doi:10.3390/foods11081068)
Supplement: Supplementary file 1 [file foods-11-01068-s001.zip › TableS2.summarized information of Iso-Seq data.pdf]

Table S2. Reads information of single-molecule long-read isoform sequencing (Iso-seq) data in sheep muscle

Data type	Total number	Min length	Max length	Average length
polymerase reads	442,966	51	363,026	97,901
Subreads	26,481,497	51	191,746	1,560
Circular Consensus Sequence (CCS) reads	331,820	55	15,574	2,119
Full-length Non-Concatemer (FLNC) reads	247,201	50	9,144	1,732
