# Supplementary material for: Preliminary Results about Lamb Meat Tenderness Based on the Study of Novel Isoforms and Alternative Splicing Regulation Pathways Using Iso-seq, RNA-seq and CTCF ChIP-seq Data
Source: Foods. 2022 Apr 7;11(8):1068. doi: 10.3390/foods11081068 (PMC9025809; doi:10.3390/foods11081068)
Supplement: Supplementary file 1 [file foods-11-01068-s001.zip › TableS7. Sunmmaried informatio of CTCF ChIP-seq data.Final.pdf]

Sample	Raw Reads	Clean Reads	Q30	mapped Reads	map Rate
IP_M045	40824586	40091240	94.44 %	38671219	96.46 %
IP_M090	65992524	64774520	93.37 %	64444293	99.49 %
In_M045	52698946	51761624	93.81 %	51577058	99.64 %
In_M090	54760180	53872826	94.31 %	53735714	99.75 %
