# Supplementary figures and images for: Preliminary Results about Lamb Meat Tenderness Based on the Study of Novel Isoforms and Alternative Splicing Regulation Pathways Using Iso-seq, RNA-seq and CTCF ChIP-seq Data
Source: Foods. 2022 Apr 7;11(8):1068. doi: 10.3390/foods11081068 (PMC9025809; doi:10.3390/foods11081068)

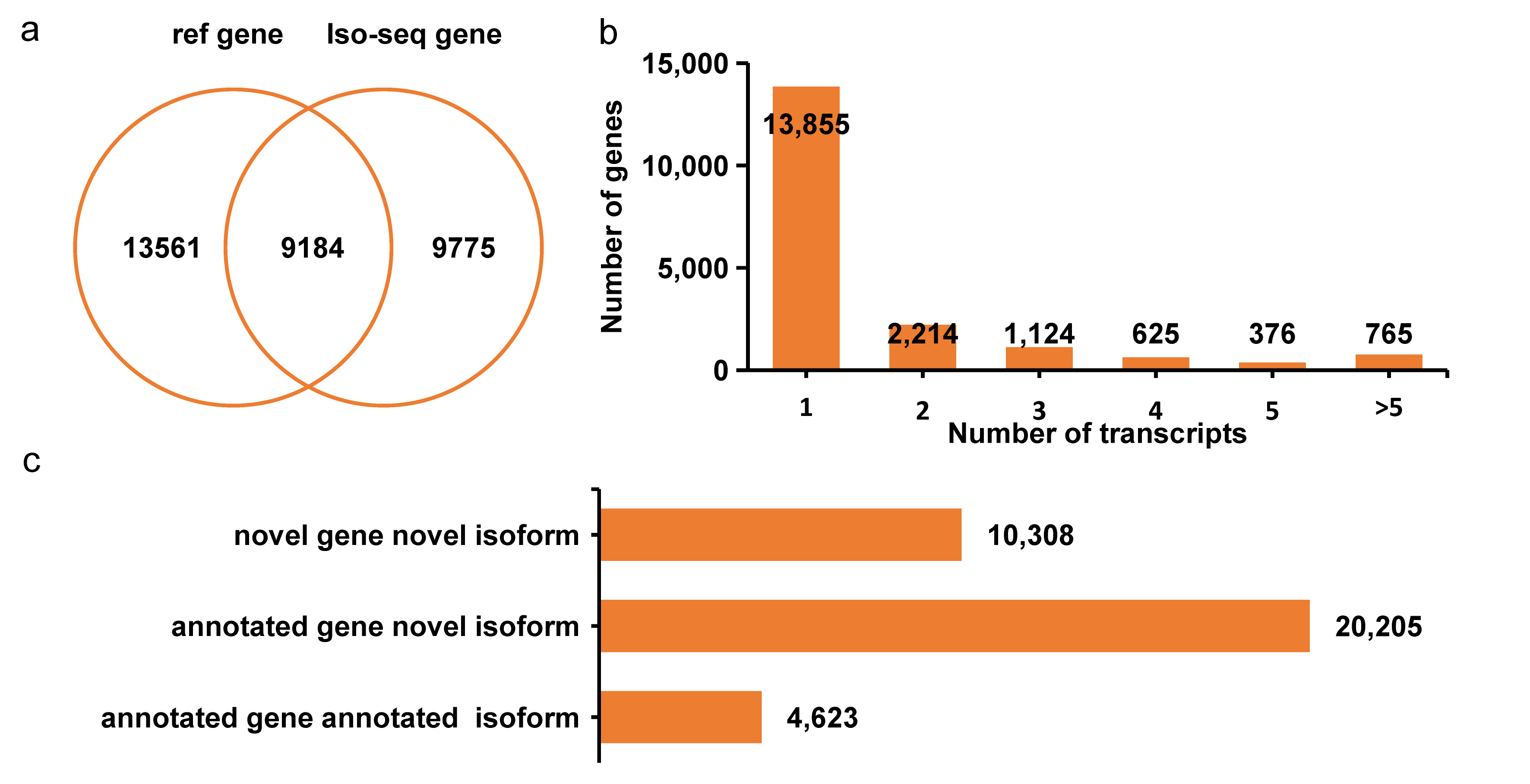

Supplement: Supplementary file 1 [file foods-11-01068-s001.zip › FigureS1.tif]

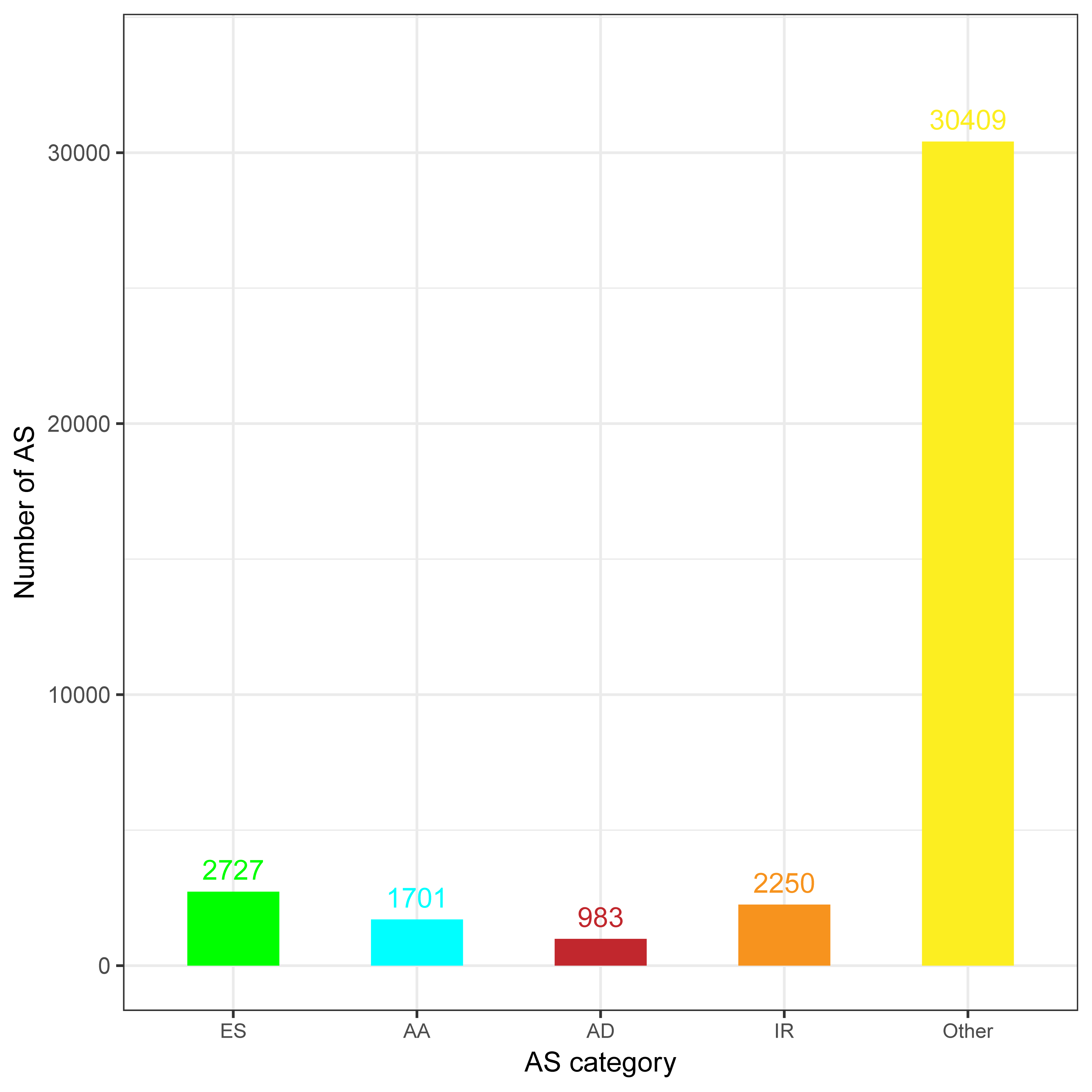

Supplement: Supplementary file 1 [file foods-11-01068-s001.zip › FigureS2.tif]
